# Mindfulness Meditation Is Related to Long-Lasting Changes in Hippocampal Functional Topology during Resting State: A Magnetoencephalography Study

**DOI:** 10.1155/2018/5340717

**Published:** 2018-12-18

**Authors:** Anna Lardone, Marianna Liparoti, Pierpaolo Sorrentino, Rosaria Rucco, Francesca Jacini, Arianna Polverino, Roberta Minino, Matteo Pesoli, Fabio Baselice, Antonietta Sorriso, Giampaolo Ferraioli, Giuseppe Sorrentino, Laura Mandolesi

**Affiliations:** ^1^Department of Motor Sciences and Wellness, University of Naples “Parthenope”, Naples, Italy; ^2^Department of Engineering, University of Naples “Parthenope”, Naples, Italy; ^3^Institute of Applied Sciences and Intelligent Systems, CNR, Pozzuoli, Italy; ^4^Department of Science and Technology, University of Naples “Parthenope”, Naples, Italy; ^5^Institute for Diagnosis and Cure Hermitage Capodimonte, Naples, Italy; ^6^IRCCS Santa Lucia Foundation, Rome, Italy

## Abstract

It has been suggested that the practice of meditation is associated to neuroplasticity phenomena, reducing age-related brain degeneration and improving cognitive functions. Neuroimaging studies have shown that the brain connectivity changes in meditators. In the present work, we aim to describe the possible long-term effects of meditation on the brain networks. To this aim, we used magnetoencephalography to study functional resting-state brain networks in Vipassana meditators. We observed topological modifications in the brain network in meditators compared to controls. More specifically, in the theta band, the meditators showed statistically significant (*p* corrected = 0.009) higher degree (a centrality index that represents the number of connections incident upon a given node) in the right hippocampus as compared to controls. Taking into account the role of the hippocampus in memory processes, and in the pathophysiology of Alzheimer's disease, meditation might have a potential role in a panel of preventive strategies.

## 1. Introduction

Several studies have shown that the constant practice of meditation induces neuroplasticity phenomena, including the reduction of age-related brain degeneration [1–3] and the improvement of cognitive functions [4]. More specifically, the effects of meditation are correlated to improvements in attention [5], working memory [6], spatial abilities [7], and long-term memory [2].

There are several meditation traditions that share some similarities but differ in practices and theoretical orientation [8]. Recently, meditation practices have been divided into two broad categories: focused attention (or concentrative style) meditation and open awareness (or mindfulness) meditation, depending on how the attentional processes are directed [9]. Concentrative meditation techniques consist in focusing on specific mental or sensory activity, such as a repeating sound, a mental image, or specific bodily sensations such as breathing. On the other hand, the fundamental feature of mindfulness meditation is a particular type of attention characterized by a nonjudgmental thinking which allows the meditator to act in the daily life in a “nonreactive” manner [2, 5].

Over the past ten years, scientific studies have begun to focus on one of the main open awareness or mindfulness meditations, named Vipassana meditation (VM) [10]. VM is a Buddhist practice that consists in focusing on the sensory awareness of the moment with a mental condition of calm and nonreactivity [11, 12]. In the ancient Indian language Pali, Vipassana means “introspection, penetrative vision, observation and understanding of reality as it is” [13]. Through the VM, one may reach meta-awareness [14], having cognition of the mental and emotional processes occurring during meditation [15].

Neuroimaging and electroencephalographic studies have shown that the brain connectivity of meditators changes as they meditate [9, 15, 16] as well as in the resting state [17–19]. Furthermore, the constant practice of meditation causes widespread long-term changes in structural connectivity [20], suggesting that meditation might induce neural plasticity.

Magnetoencephalography (MEG) has been used to study the brain activity since it directly captures the neuronal oscillations [21]. While retaining the high temporal resolution of the EEG, MEG signals are not distorted by the layers surrounding the brain, allowing for a temporally and spatially precise reconstruction of the neural activity within the brain [22]. Using MEG, Dor-Ziderman et al. have documented a correlation between the distinct type of self-awareness, reached through mindfulness meditation, and the power decrease in gamma and beta bands in several specific brain areas [23, 24]. Furthermore, MEG (as well as EEG) signals allow the use of the phase in order to quantify synchronization between brain areas [25].

In recent years, a graph theoretical approach has been applied to functional signals in order to extract relevant features of the interactions among brain areas [26]. Applying network theory to MEG signals, Marzetti et al. have shown that meditation induces a coupling of the posterior cingulate cortex to nodes of the Default Mode Network and of the executive control brain network in the alpha frequency band [27]. More recently, in an EEG study, it has been shown that meditation is associated with increased brain network integration [28].

Despite the well-documented role of the meditative practice on brain functions, little attention has been granted to the long-lasting effects of meditative practice on synchronization between neuronal ensembles and on brain topology. To the best of our knowledge, there are no source-level MEG studies that have investigated the brain network synchronization and its topological features in relation to meditative practice.

In this work, we hypothesized that the routine practice of mindfulness meditation may induce long-lasting effects in the topology of brain areas involved in attention and/or memory. To test our hypothesis, we have considered the sources within the brain as nodes and the functional interactions between them as links. We based the link estimation on the Phase Lag Index (PLI), which quantifies phase synchronization between cerebral areas [29]. Subsequently, we obtained the minimum spanning tree (MST), which allows the construction of a unique subgraph that connects all the anatomical nodes, identifying the backbone of the network [30]. We applied such analysis to a cohort of routinely Vipassana meditators and to controls (people with no experience in meditation) in the resting-state condition, comparing their brain network topology.

## 2. Methods

### 2.1. Participants

Twenty-nine people who had been practicing VM for more than one year and 31 people who had never meditated in their life were recruited for the study. The meditators were from a Vipassana community located in Caivano (Naples). All participants had been meditating in the last year for 1 h or more, for at least five days a week.

Criteria of inclusion were the following: (1) no major internal illnesses, (2) no neurological or psychiatric illnesses, (3) left dominance, and (4) participation in Vipassana retreats in the last year. Three meditators were rejected: one left-handed, one presenting a neurological ailment, and one with insufficient quality of the MEG signals. Two controls were discarded due to excessive signal noise.

The final Vipassana meditator group was composed of twenty-six participants (eight males and eighteen females), mean age 42.6 years (±SE 2.4), mean years of meditation experience 6.41 (±SE 1.4). The final control group was composed of twenty-nine (nine males and twenty females) age-gender-education-race-matched controls, mean age 43 (±SE 1.98), no experience in meditation. All participants were native Italian speakers (Table 1).

This study was carried out in accordance with the recommendations of Comitato Etico Campania Centro. The protocol was approved by the “Comitato Etico Campania Centro” (Prot.n.93C.E./Reg. n.14-17OSS). All subjects gave written informed consent in accordance with the Declaration of Helsinki.

### 2.2. MEG Acquisition

**Figure 1:**
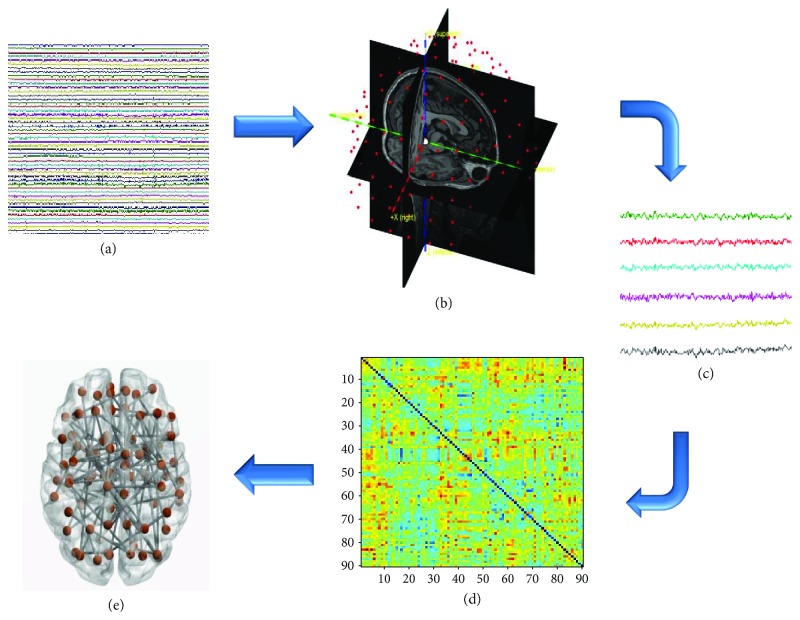
Schematic illustration of the reconstruction of MEG signals. (a) MEG signals. (b) Beamforming based on a template MRI. (c) Reconstructed time series for 90 regions of interest (ROIs). (d) Connectivity matrix containing the functional connections between ROIs based on the PLI. (e) Construction of the MST-based brain network (where each ROIs is a node and each functional connection an edge).

The MEG system was developed by the National Research Council, Pozzuoli, Naples, at the Institute of Applied Sciences and Intelligent Systems “E. Caianiello.” The system is equipped with 163 magnetometers, including 9 reference sensors that are located further away from the helmet [31], and it was placed in a magnetically shielded room (ATB, Ulm, Germany) to reduce background noise. Acquisition, preprocessing, and source reconstruction were carried out as previously described [32]. More in details, before the acquisition, four positions coils were placed on the subject's head and were digitalized using Fastrak (Polhemus®). The coils were activated, and localized, at the beginning of each segment of registration. The subject was seated on a comfortable armchair placed in the shielded room. Electrocardiographic and electrooculographic signals were corecorded to aid artefact removal [33]. The brain activity (Figure 1(a)) was recorded during resting state for two distinct segments of 2.5 minutes with the eyes closed. MEG data, after an antialiasing filter, were acquired with a sampling frequency of 1024 Hz. The signal was then filtered using a fourth order Butterworth IIR band-pass filter in the 0.5–48 Hz band.

### 2.3. Preprocessing

Firstly, Principal Component Analysis (PCA) was used to reduce environmental noise [34, 35]. Specifically, the filter is obtained by orthogonalizing the reference signals to obtain a base, projecting the brain sensors on the base of the noise and removing the projections to obtain clean data [35]. We adopted the PCA filtering implementation available within the Fieldtrip toolbox [36]. Subsequently, noisy channels were removed manually through visual inspection by an experienced rater [33]. Finally, for each subject, supervised Independent Component Analysis (ICA) [37] was used to remove physiological, cardiac (generally one component), and blinking (if present) artefacts from the MEG signals.

The first ten epochs of 8 seconds for each subject that did not contain artefacts (either system related or physiological) or strong environmental noise were selected. The length of 8 seconds is a trade-off between the need to have enough cleaned epochs, to avoid drowsiness [33] and to obtain a reliable estimate of the connectivity measure [38].

### 2.4. Source Reconstruction

All the processing related to the beamforming procedure has been done using the Fieldtrip toolbox [36]. Based on a MRI template, the volume conduction model proposed by Nolte [39] was considered and the Linearly Constrained Minimum Variance (LCMV) beamformer [40] was implemented to reconstruct time series related to the centroids of 116 regions-of-interest (ROIs), derived from the Automated Anatomical Labeling (AAL) atlas [41–43] (Figure 1(b)). Although cerebellar areas have been reconstructed previously in some EEG studies [44, 45], we decided not to include them (hence taking into account 90 ROIs) given their lower reliability [46, 47]. For each source, we projected the time series along the dipole direction that explains the most variance by means of singular value decomposition (SVD). Source time series were resampled at 512 Hz (Figure 1(c)).

### 2.5. Connectivity and Network Analysis

The PLI was used to estimate functional connectivity [29], using BrainWave software (CJS, version 09.152.1.23, available from http://home.kpn.nl/stam7883/brainwave.html). The PLI is based on the distribution of the differences of the instantaneous phases (*∆*Φ(*t*)) (derived from the Hilbert transform of the time series) and for two-time series and is computed as

(1) $$\begin{array}{c} PLI=\left|\langle \mathrm{sign}\left[\sin\left(\Delta \Phi \left(t_{k}\right)\right)\right]\rangle \right|, \end{array}$$

where “< >” indicates the mean value, “sign” stands for the signum function, “|.|” denotes the absolute value and “*t*_*k*_” are the samples. The phase difference is defined in the [−*π*, *π*] range. This measure is insensitive to volume conduction (at the cost of discarding true zero–lag interactions). PLI values range between 0 and 1, where 1 indicates perfect synchronization and 0 indicates nonsynchronous activity. We obtained a 90 × 90 adjacency matrix for each epoch for each subject, in all the frequency bands [29]. For each epoch, the PLI matrix was computed, and after this step, they were merged by arithmetic average. Hence, by computing the PLI for each couple of brain regions, we obtained a 90 × 90 weighted adjacency matrix (Figure 1(d)) for each epoch and for each subject, in all of the frequency bands: delta (0.5–4.0 Hz), theta (4.0–8.0 Hz), alpha (8.0–13.0 Hz), beta (13.0–30.0 Hz), and gamma (30.0–48.0 Hz).

The weighted adjacency matrix was used to reconstruct a network, where the 90 areas of the AAL atlas are represented as nodes and the PLI values form the weighted edges. A frequency-specific minimum spanning tree was calculated for each epoch (Figure 1(e)). Since we were interested in the strongest connections, for the construction of the MST, the edge weight was defined as 1/PLI. In fact, Kruskal's algorithm [48] first ranks the links in ascending order and then constructs the network by adding one link at a time, discarding links that would form a loop. The algorithm proceeds until all nodes are connected resulting in a loopless graph with *N* nodes and *M* = *N* − 1 links.

To avoid some of the biases in traditional network analyses [49], we used the minimum spanning tree (MST) that allows for an unbiased topological interpretation of the results [50, 51]. Based on the MST, we calculated both global (leaf fraction, degree divergence, and tree hierarchy) and nodal parameters (degree, betweenness centrality, and eccentricity) [51, 52]. The leaf fraction is defined as the fraction of nodes with a degree of 1 [52], providing an indication of the integration of the network. A higher leaf fraction implies that the network tends toward a starlike topology, where the nodes are on average closer to each other as compared to a more line-like topology. The degree divergence is a measure of the broadness of the degree distribution, related to the resilience against attacks [51]. Finally, the tree hierarchy is defined as the number of leaf over the maximum betweenness centrality. The idea behind this measure is that an optimal network should achieve efficient communication while avoiding hub overload. The tree hierarchy has been designed to quantify the balance between both features.

Furthermore, we calculated the degree, the betweenness centrality (BC), and the eccentricity for each node [53], in order to determine if specific regions differed according to the number of years of meditation. The degree is the number of connection incident on a given node. The BC is defined as the number of the shortest paths passing through a given node over the total of the shortest paths of the network [53]. The eccentricity is defined as the longest path between a node and any other node of the network. The lower the eccentricity, the more central the node is [51]. These metrics were calculated for each epoch and subsequently averaged across epochs for each subject separately.

### 2.6. Statistical Analysis

All statistical analyses were performed in Matlab (Mathworks®, version R2013a). To compare the two groups, for each frequency band and for each parameter, we used permutation testing [54] where the null distribution for between-group differences is derived from the data. In details, assuming no group differences, the labels of the subjects were permuted 10,000 times. Each time, the difference between the averages of the two groups was computed, obtaining the null distribution for between-group differences. Such distribution was used to define the statistical significance of the observed difference between the two groups. We used the False Discovery Rate (FDR) [55] to correct for multiple comparisons. A significance level of *p* ≤ 0.05 was used.

## 3. Results

After building a PLI-based adjacency matrix, we used an MST-based network approach in order to compare nodal and global topological parameters in meditators and controls.

Global parameters (leaf fraction, degree divergence, and tree hierarchy) were not significantly different between groups before correction for multiple comparisons between metrics.

With regard to nodal metrics, before correction for multiple comparisons, we found differences between meditators and controls in regions that are typically affected by the meditative practice. In particular, in the amygdala, in the gamma band, both the degree and BC were higher in meditators as compared to controls (*p* = 0.049, *p* = 0.034, respectively). The caudatus, another area known to be affected by VM, was shown to be more connected in the theta band in meditators (*p* = 0.0049). In the prefrontal cortex, we found fairly widespread differences in areas such as (among others) the gyrus rectus in the alpha band (*p* = 0.0201) and the cingulum in the theta band (*p* = 0181). The calcarine cortex appeared less connected in the theta band (*p* = 0.011) and more connected in the gamma band (*p* = 0.038). For the reader's interpretation, we report in Table 2 the uncorrected *p* values. However, it is important to notice that these results are not FDR corrected and should be considered with extreme caution.

**Figure 2:**
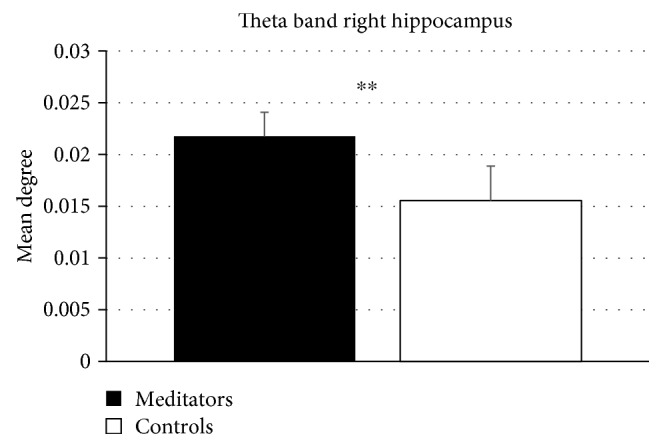
Mean degree in the theta band in the right hippocampus. Data are expressed as mean ± SD. The asterisk indicates statistical significance *p* = 0.009.

After correction for multiple comparisons between areas, higher degree (*p* corrected = 0.009) was found in meditators, in the theta band, in the right hippocampus (Figure 2). No other nodal significances held FDR in any other frequency bands.

## 4. Discussion

The present study was addressed to verify whether mindfulness meditation may induce long-lasting effects in topological features in brain networks. To this aim, we applied network analysis to compare source-level MEG data obtained from a cohort of routinely Vipassana meditators to controls, during resting-state condition. No difference in the global metrics was observed. Moreover, before FDR correction, we observed altered topology in meditators in a number of brain areas, including the frontal lobes, the temporal lobes, the occipital lobes, the amygdala, and the caudatus. Importantly, after FDR correction, our results showed that the right hippocampus displays a higher degree in the theta band with respect to controls.

As expected, we did not find difference in the global metrics comparing meditators to controls. Global metrics capture widespread reorganization in the brain activity, and those are found to be altered typically in processes that cause diffuse damage to the brain, such as neurological diseases [32, 56–58]. Our results showed that the connectedness or the brain network (Leaf fraction), its scale-freeness (Degree divergence), and its resiliency versus targeted attacks (Tree hierarchy) are not affected by VM. Hence, we conclude that meditation does not induce a reorganization in the whole network.

When considering nodal results not corrected for multiple comparisons, according to the previous literature, we observed areas with altered topology in the frontal lobes [18, 59], in the temporal lobes [16, 60], in the amygdala [61], in the caudatus [19], and in the occipital lobes [62] in multiple frequency bands. The results we found are in multiple frequency bands, which is in line with the previous literature taken altogether [9, 63–65]. However, provided the lack of correction for multiple comparisons, such results are only explorative.

After correction for multiple comparisons between areas, the right hippocampus of meditators showed a higher degree in the theta band. This result is in line with previous evidence showing the central role of the hippocampus in meditative practices [10, 66, 67]. The higher degree implies that meditators have more link incident upon the right hippocampus as compared to controls. This can be interpreted as the right hippocampus being more connected to the rest of the network. More precisely, this is to say that the topology of the area (i.e., the properties of its logical relationships with the rest of the brain) is modified so that the role of that area is more prominent within the network. Provided we are exploring the long-term effects of meditation, one would expect that the areas that are involved more (and more often) in the meditative practice would be the ones undergoing long-lasting functional modifications. According to this evidence, it has been observed that meditators show a larger volume [67] and greater grey matter concentration [68] in the right hippocampus as compared to nonmeditators. Interestingly, the connectivity of the same area [60] is increased during the practice of meditation as well. Such results might be in line with our finding, showing that long-term changes are induced by VM in the right hippocampus.

Our results were specific to the right hippocampus. This might be related to the different functional specialization between the right and left hippocampus. It was observed that while the left hippocampus was activated both in the construction of past events and in the future ones, the right hippocampus was activated only during the creation of future events (prospective memory) [69]. It has been reported that, when new information is being processed, the synchronization between the hippocampus and the prefrontal cortex happens mainly in the theta band [70, 71]. More in detail, during the imagination of future events, novel memory and new elements are learned and prospective memory is built. Besides the involvement in prospective memory, the right hippocampus is also implicated in visuo-spatial memory processes [72–74], with a critical role in the consolidation processes.

Interestingly, we observed that the difference in the centrality of the hippocampus was specific to the theta band, which, as mentioned above, is involved in memory processes [75]. According to Buzsáki, theta oscillations are considered as an “essential temporal organizer,” helping the orientation in time during episodic memory as well as in the physical world [76]. Increases in the theta power during meditative practice have been widely reported [77, 78] also in the temporal areas [79].

In the light of the functional role of the right hippocampus and its activation in the theta band, our results indicate that meditation might have functional effects on prospective and spatial memories. These findings suggest that meditation, if constantly practiced, could be a candidate as a nonpharmacological intervention in pathologies characterized by alteration in the hippocampal areas.

It should finally be noted that in the literature there are often conflicting data. This may be explained by the different methodological approaches, such as the analysis of the meditative phase vs. resting state, the type of meditation explored (focused attention vs. mindfulness meditation), and finally, the modality of data acquisition and/or data analysis. Of note, our study is the only study to date which has analysed the difference of the brain network between Vipassana meditators and controls during the resting state and using source-level magnetoencephalography.

Our results seem to bear a promise of what the effects of meditation might be on brain networks. Nevertheless, it is important to underline that they are preliminary and that further analyses will have to be carried out. For example, it is essential to investigate perspective and spatial memory in meditators and nonmeditators during tasks in order to relate the functional connectivity with cognitive functioning. Furthermore, it will be helpful to analyse the brain networks in people that practice other forms of meditation, in order to better characterize the benefits of specific meditative practices.

Despite the aforementioned limits, we showed that routine meditative practice is associated with a long-lasting change in the topology of definite brain areas, suggesting that meditation might be able to induce brain plasticity.

## Figures and Tables

**Table 1:** Cohort characterization. Means and standard error are reported.

Features	Size	Gender (M/F)	Age	Education	Years of meditation	Hours of meditation/day
Groups						
Meditators	26	8/18	42.30 ± 2.4	15.12 ± 0.66	6.4 ± 1.4	1.33 ± 0.72
Controls	29	9/20	43 ± 1.98	15.72 ± 0.70	—	—

**Table 2:** Uncorrected *p* values comparing nodal metrics between meditators and controls. “+” indicates that meditators display higher values as compared to controls, “-” signifies the opposite.

	Degree					Betweenness centrality					Eccentricity				
	Delta	Theta	Alpha	Beta	Gamma	Delta	Theta	Alpha	Beta	Gamma	Delta	Theta	Alpha	Beta	Gamma
Frontal_Inf_Orb_L	0.0055-	n.s.	n.s.	n.s.	n.s.	n.s.	n.s.	n.s.	0.0097-	n.s.	n.s.	n.s.	n.s.	n.s.	n.s.
Frontal_Sup_Orb_L	n.s.	n.s.	n.s.	n.s.	n.s.	0.0415+	n.s.	n.s.	n.s.	n.s.	n.s.	n.s.	n.s.	n.s.	n.s.
Frontal_Mid_Orb_L	n.s.	n.s.	n.s.	n.s.	0.0263-	n.s.	n.s.	n.s.	n.s.	0.0319-	n.s.	n.s.	n.s.	n.s.	n.s.
Cingulum_Ant_L	n.s.	0.0337-	n.s.	n.s.	n.s.	n.s.	n.s.	n.s.	0.0118+	n.s.	n.s.	n.s.	n.s.	n.s.	n.s.
Cingulum_Mid_L	0.0181-	n.s.	n.s.	n.s.	n.s.	n.s.	n.s.	n.s.	n.s.	n.s.	n.s.	n.s.	n.s.	n.s.	n.s.
Olfactory_L	n.s.	n.s.	n.s.	n.s.	0.0292-	n.s.	n.s.	n.s.	n.s.	n.s.	n.s.	n.s.	n.s.	n.s.	n.s.
Frontal_Sup_L	n.s.	n.s.	n.s.	0.0239-	n.s.	n.s.	n.s.	n.s.	n.s.	n.s.	n.s.	n.s.	n.s.	n.s.	n.s.
Frontal_Sup_Medial_L	n.s.	n.s.	0.0044-	n.s.	n.s.	n.s.	n.s.	n.s.	n.s.	n.s.	n.s.	n.s.	n.s.	n.s.	n.s.
Frontal_Med_Orb_R	n.s.	n.s.	n.s.	n.s.	0.0338+	n.s.	n.s.	n.s.	n.s.	n.s.	n.s.	n.s.	n.s.	n.s.	n.s.
Rectus_R	n.s.	n.s.	n.s.	n.s.	n.s.	n.s.	n.s.	0.0201+	n.s.	n.s.	n.s.	n.s.	n.s.	n.s.	n.s.
Temporal_Pole_Mid_L	n.s.	n.s.	0.0456+	n.s.	n.s.	n.s.	n.s.	0.0085+	n.s.	n.s.	n.s.	n.s.	n.s.	n.s.	n.s.
Heschl_L	n.s.	n.s.	n.s.	0.0102+	n.s.	n.s.	n.s.	n.s.	n.s.	n.s.	n.s.	n.s.	n.s.	n.s.	n.s.
Hippocampus_L	n.s.	n.s.	n.s.	n.s.	0.0161-	n.s.	n.s.	n.s.	n.s.	0.0472-	n.s.	n.s.	n.s.	n.s.	n.s.
Amygdala_L	n.s.	n.s.	n.s.	n.s.	0.0495+	n.s.	n.s.	n.s.	n.s.	0.0347+	n.s.	n.s.	n.s.	n.s.	n.s.
Temporal_Pole_Mid_R	n.s.	n.s.	n.s.	n.s.	n.s.	0.0411+	n.s.	n.s.	n.s.	0.0183-	n.s.	n.s.	n.s.	n.s.	n.s.
Temporal_Sup_R	0.0064+	n.s.	n.s.	n.s.	n.s.	n.s.	n.s.	n.s.	n.s.	n.s.	n.s.	n.s.	n.s.	n.s.	n.s.
Temporal_Inf_R	n.s.	n.s.	n.s.	n.s.	0.0115+	n.s.	n.s.	n.s.	n.s.	n.s.	n.s.	n.s.	n.s.	n.s.	n.s.
ParaHippocampal_R	n.s.	n.s.	n.s.	n.s.	0.0318+	n.s.	n.s.	n.s.	n.s.	n.s.	n.s.	n.s.	n.s.	n.s.	n.s.
Hippocampus_R	n.s.	0.001+	n.s.	n.s.	n.s.	n.s.	0.0081+	n.s.	0.0029+	n.s.	n.s.	n.s.	n.s.	n.s.	n.s.
Insula_R	n.s.	n.s.	n.s.	n.s.	n.s.	n.s.	n.s.	n.s.	n.s.	n.s.	n.s.	n.s.	0.0483+	n.s.	n.s.
Occipital_Sup_L	n.s.	n.s.	n.s.	0.0357+	n.s.	n.s.	n.s.	n.s.	n.s.	n.s.	n.s.	n.s.	n.s.	n.s.	n.s.
Occipital_Mid_L	n.s.	n.s.	n.s.	n.s.	n.s.	0.0155-	n.s.	n.s.	n.s.	n.s.	n.s.	n.s.	n.s.	n.s.	n.s.
Cuneus_L	n.s.	n.s.	n.s.	0.0062+	n.s.	n.s.	n.s.	0.007-	n.s.	n.s.	n.s.	n.s.	n.s.	n.s.	n.s.
Calcarine_L	n.s.	n.s.	n.s.	n.s.	n.s.	n.s.	0.0367-	n.s.	n.s.	n.s.	n.s.	n.s.	n.s.	n.s.	n.s.
Occipital_Mid_R	n.s.	n.s.	n.s.	0.0289+	n.s.	n.s.	0.0418+	n.s.	n.s.	n.s.	n.s.	n.s.	n.s.	n.s.	n.s.
Occipital_Sup_R	n.s.	n.s.	n.s.	n.s.	n.s.	0.0138-	n.s.	n.s.	n.s.	n.s.	n.s.	n.s.	n.s.	n.s.	n.s.
Cuneus_R	n.s.	n.s.	n.s.	n.s.	n.s.	0.03800-	n.s.	n.s.	n.s.	n.s.	n.s.	n.s.	n.s.	n.s.	n.s.
Calcarine_R	0.0115-	n.s.	n.s.	n.s.	n.s.	n.s.	n.s.	n.s.	n.s.	0.0381+	n.s.	n.s.	n.s.	n.s.	n.s.
SupraMarginal_R	n.s.	n.s.	n.s.	n.s.	n.s.	0.0034+	n.s.	n.s.	n.s.	n.s.	n.s.	n.s.	n.s.	n.s.	n.s.
Parietal_Inf_R	n.s.	n.s.	n.s.	n.s.	0.004+	n.s.	n.s.	n.s.	n.s.	n.s.	n.s.	n.s.	n.s.	n.s.	n.s.
Pallidum_L	0.0434-	n.s.	n.s.	n.s.	n.s.	n.s.	n.s.	n.s.	n.s.	n.s.	n.s.	n.s.	n.s.	n.s.	n.s.
Caudate_L	n.s.	0.0049+	n.s.	n.s.	n.s.	n.s.	n.s.	n.s.	n.s.	n.s.	n.s.	n.s.	n.s.	n.s.	n.s.
Putamen_R	n.s.	n.s.	n.s.	n.s.	n.s.	n.s.	n.s.	n.s.	n.s.	0.0221+	n.s.	n.s.	n.s.	n.s.	n.s.

## Data Availability

The data used to support the findings of this study are available from the corresponding author upon request.
